# Zebrafish modeling of intestinal injury, bacterial exposures and medications defines epithelial *in vivo* responses relevant to human inflammatory bowel disease

**DOI:** 10.1242/dmm.037432

**Published:** 2019-08-13

**Authors:** Ling-shiang Chuang, Joshua Morrison, Nai-yun Hsu, Philippe Ronel Labrias, Shikha Nayar, Ernie Chen, Nicole Villaverde, Jody Ann Facey, Gilles Boschetti, Mamta Giri, Mireia Castillo-Martin, Tin Htwe Thin, Yashoda Sharma, Jaime Chu, Judy H. Cho

**Affiliations:** 1 Department of Genetics and Genomic Sciences, Icahn School of Medicine at Mount Sinai, New York, NY 10029, USA; 2 The Charles Bronfman Institute for Personalized Medicine, Icahn School of Medicine at Mount Sinai, New York, NY 10029, USA; 3 Department of Pediatrics, Icahn School of Medicine at Mount Sinai, New York, NY 10029, USA; 4 Department of Oncological Science, Icahn School of Medicine at Mount Sinai, New York, NY 10029, USA; 5 Departments of Pathology, Icahn School of Medicine at Mount Sinai, New York, NY 10029, USA

**Keywords:** Crohn's disease, DSS injury model, Epithelial barrier, IBD, Lysosome-rich enterocytes

## Abstract

Genome-wide association studies have identified over 200 genomic loci associated with inflammatory bowel disease (IBD). High-effect risk alleles define key roles for genes involved in bacterial response and innate defense. More high-throughput *in vivo* systems are required to rapidly evaluate therapeutic agents. We visualize, in zebrafish, the effects on epithelial barrier function and intestinal autophagy of one-course and repetitive injury. Repetitive injury induces increased mortality, impaired recovery of intestinal barrier function, failure to contain bacteria within the intestine and impaired autophagy. Prostaglandin E2 (PGE2) administration protected against injury by enhancing epithelial barrier function and limiting systemic infection. Effects of IBD therapeutic agents were defined: mesalamine showed protective features during injury, whereas 6-mercaptopurine displayed marked induction of autophagy during recovery. Given the highly conserved nature of innate defense in zebrafish, it represents an ideal model system with which to test established and new IBD therapies targeted to the epithelial barrier.

This article has an associated First Person interview with the first author of the paper.

## Abstract (precis)

**Summary:** As IBD attempts to enter the era of precision medicine, our models provide an opportunity for the rapid and sophisticated determination of time course, cellular contributions and mechanisms of new drugs.

## INTRODUCTION

IBD is classified into two subtypes: Crohn's disease (CD) and ulcerative colitis (UC). While UC affects the superficial layers of the intestine, CD often affects deeper layers of the bowel wall. The most significant loci identified in genome-wide association studies (GWAS) for IBD include protein-altering alleles in *IL23R* (interleukin 23 receptor), *NOD2* (nucleotide oligomerization domain 2), *ATG16L1* (autophagy-related 16-like 1) (Huang et al., 2017), *LRRK2* (leucine-rich repeat kinase 2) (Hui et al., 2018) and *CSF2RB* (colony stimulating factor 2 receptor) (Chuang et al., 2016), as well as non-coding risk alleles near *PTGER4* (prostaglandin E receptor 4), that increase gene expression (Libioulle et al., 2007; Jostins et al., 2012; Liu et al., 2015). The *NOD2* risk alleles show impaired intracellular sensing of bacterial peptidoglycan (Bonen et al., 2003), resulting in impaired NF-κB activation (Ogura et al., 2001). The *ATG16L1* alanine risk allele at codon 300 increases its proteolytic cleavage by caspase 3 and 7 (Lassen et al., 2014; Murthy et al., 2014), implicating impaired autophagy in CD.

The striking overlap between genes implicated in CD and mycobacterial susceptibility highlights a key role for innate defense and containment of microbes (Jostins et al., 2012). Recent literature has defined the particular value of zebrafish in modeling innate defense and immunity (Madigan et al., 2017; Cambier et al., 2017; Cronan et al., 2016). Because of their transparency over the initial weeks of life, zebrafish serve as a powerful tool for performing live *in vivo* imaging of intestinal macrophages and epithelial responses to injury and bacterial exposure.

## RESULTS

### Repeated DSS treatments prevent recovery of acidified-lysosome and mucin loss

**Fig. 1.**
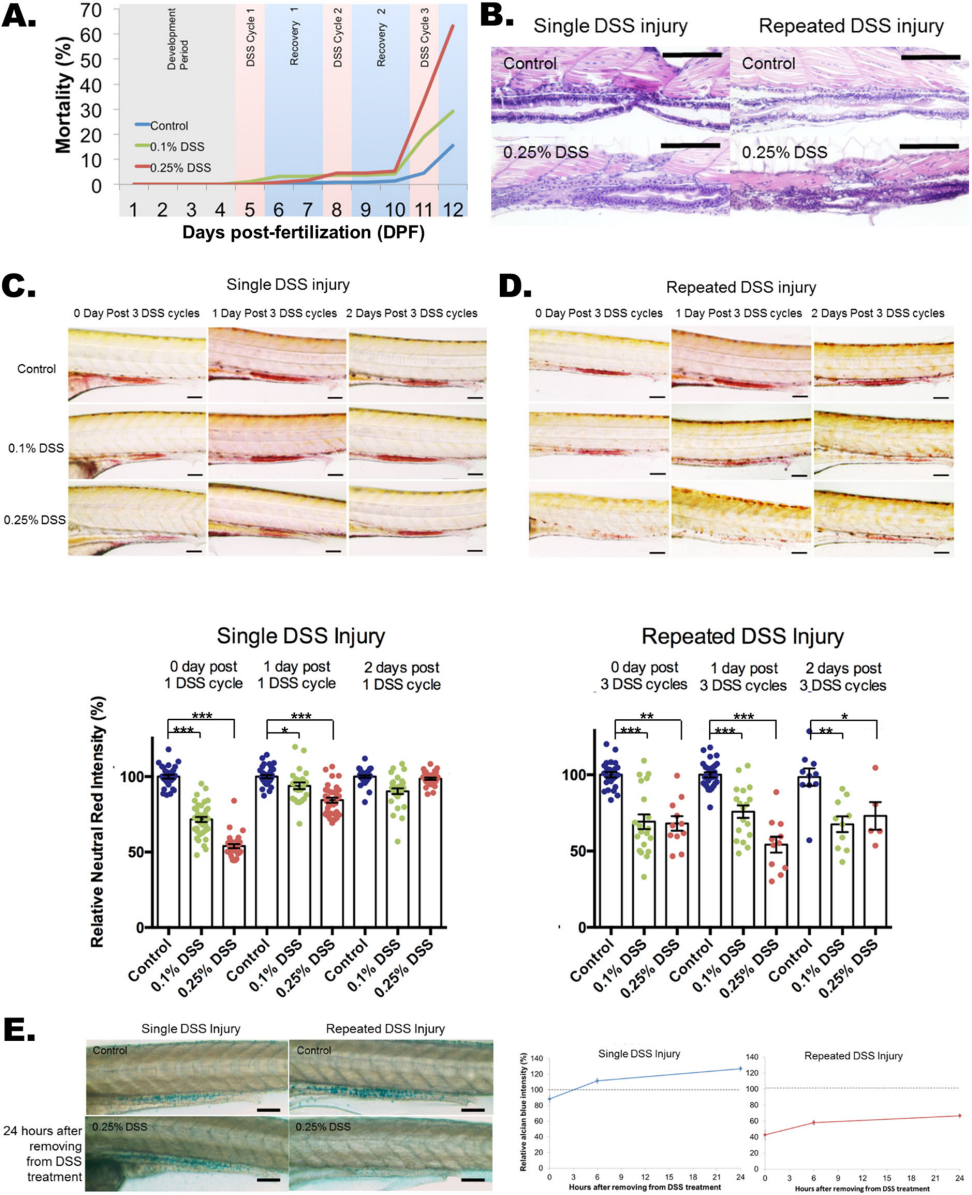
**Impaired recovery of acidic-lysosome function and mucin production after repeated DSS injury in zebrafish intestines.** (A) Dose-dependent mortality observed after repeated DSS injury (*N*=1739 from 11 clutches). Compared to the single DSS injury, which has a 98-100% survival rate (day 6), repeated DSS injury induces a high mortality rate of 63% in 0.25% DSS and 29% in 0.1% DSS. (B) Hematoxylin and eosin (H&E) staining of a longitudinal section of zebrafish larvae intestine. A total of 20 larvae per experimental condition were embedded in paraffin and sectioned at 10 μm per slide. Scale bars: 100 μm. (C,D) Quantification of Neutral Red images (top panels) and Neutral Red accumulation (bottom panel) for single (C) and repeated (D) DSS-injured zebrafish. Neutral Red accumulation in the intestine indicates normally functioning acidic lysosomes. The damage to lysosomal function is fully recovered within 2 days with the single DSS injury (C; *N*=253 from four clutches) but is impaired with the repeated DSS injury (D; *N*=137 from three clutches; day 2 *N*=5 out of 13 from single clutch due to high mortality rate of 61.5%). Neutral Red images for single (C) and repeated (D) injury are shown as control, and 0.1% and 0.25% (w/v) DSS treatments at treatment times of 1 day and 2 days after injury. Scale bars: 100 µm. ***P<0.001, **P<0.01, *P<0.05. Error bars, mean±s.e.m. (E) Images (left panel) and quantification (right panel) of Alcian Blue staining of intestine with single and repeated injury with control and 24 h after removal from DSS treatment. The quantification of total Alcian Blue intensity from single (*N*=160 from three clutches) and repeated (*N*=154 from three clutches) injury is shown. There was an increase in mucin production after single DSS injury, but mucin production was impaired after repeated DSS injury. Scale bars: 100 µm.

To model the chronicity of IBD, we first compared the effects of single- and three-course dextran sodium sulfate (DSS) injury (Fig. 1A, Fig. S1A) in zebrafish. Similar to DSS injury in mice (Chassaing et al., 2014), the gut length is significantly shortened in DSS-injured zebrafish, with no difference in the total length observed (Fig. S1B,C). Using doses as previously reported (Oehlers et al., 2012, 2013), we observed a dose-dependent induction of mortality with repeated injury (Fig. 1A; 68% for 0.25% DSS, 29% for 0.1%). With both single and repeated DSS injury, the goblet cells were observed by H&E histology (Fig. 1B).

We next sought to examine altered epithelial phenotypes contributing to the high mortality with repetitive DSS injury. Neutral Red is a dye that can diffuse across cellular membranes at physiological pH (pH=7), but is protonated under acidic conditions, at which point the dye appears red and is unable to leave the acidic compartments it has entered. Owing to the acidic nature of lysosomes, Neutral Red will accumulate in properly acidified lysosomes (Fig. S2A,C) (Oehlers et al., 2012; Chazotte, 2011), with a marked enrichment in lysosome-rich enterocytes (LREs) in the posterior mid-intestine (Fig. S2D,E) (Rodriguez-Fraticelli et al., 2015; Oehlers et al., 2011; Farber et al., 2001; Wallace et al., 2005; Rombout et al., 1985). LREs are a population of highly endocytic enterocytes in the zebrafish posterior midgut (Rodriguez-Fraticelli et al., 2015). The intensity of Neutral Red staining indicates the amount of healthy lysosomes with acidic pH (Chazotte, 2011). Therefore, a decrease in Neutral Red intensity indicates a depletion of healthy, properly acidified lysosomes. We observed a marked decrease in Neutral Red intensity with a single course of DSS that was significantly restored 1 day after DSS removal and largely recovered by 2 days after DSS removal (Fig. 1C, Fig. S2F). In contrast, with repetitive DSS injury, the reduction in Neutral Red staining observed after the third course of DSS did not recover at 1 or 2 days following DSS removal (Fig. 1D). Moreover, by quantitative real-time PCR (qPCR), we show that expression of *tnfa* and *Il1b*, encoding proinflammatory cytokines, was predominately induced in the intestine with single DSS injury, but their expression is also present in non-intestinal tissues after repeated injury (Fig. S3A,B). Similarly, Alcian Blue staining, which measures epithelial mucus production (Fig. S4A,B) (Oehlers et al., 2012), demonstrated a normal amount of mucus with no changes in goblet cell number with single-course DSS injury. However, repeated injury resulted in decreased mucus levels that did not recover when compared to controls (Fig. 1E, Fig. S5A-D).

### DSS injury with bacterial exposure impacts LRE autophagy and bacterial protein uptake

**Fig. 2.**
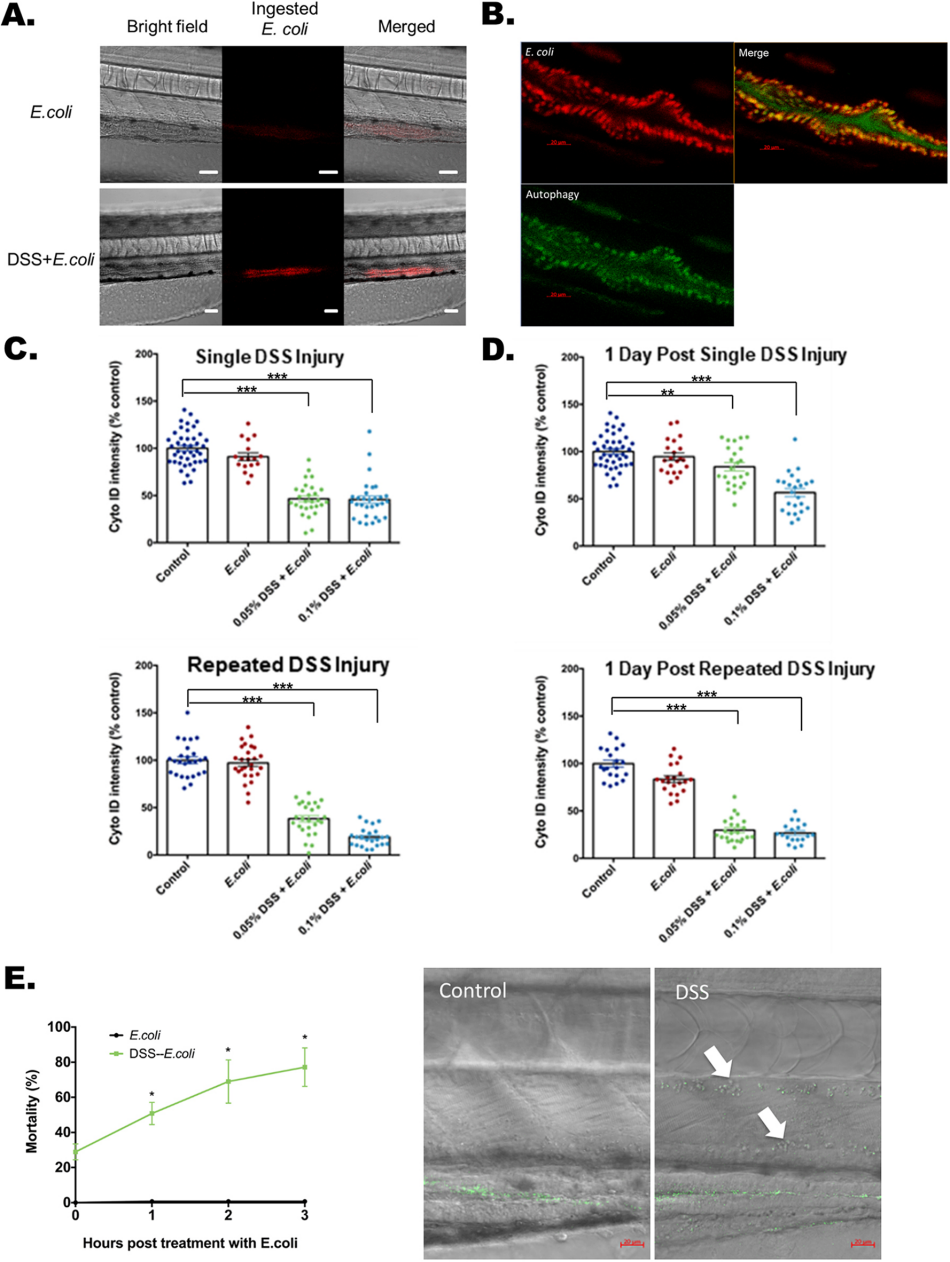
**Lysosome-rich enterocyte-mediated bacterial protein uptake and autophagy with injury and bacterial exposure.** (A) Bright-field, fluorescent and merged images of pHrodo-labeled heat-killed *E. coli* K12 MG1655 within a zebrafish larval intestine with and without DSS treatment. A total of 20 larva from each experimental condition were treated with the pHrodo-labeled *E. coli* proteins. The pHrodo dye is a pH-sensing dye indicating ingested *E. coli* proteins. Scale bars: 50 μm. The quantification is shown at the 0 h time point of Fig. 3G. (B) Uptake of *E. coli* proteins (red) colocalized with Cyto-ID-positive autophagosomes (green) in intestine. Scale bars: 20 μm. (C) Quantification of Cyto-ID intensities immediately following single (top panel) and repeated DSS injury (bottom panel). DSS [0.05% and 0.1% (w/v)] was applied at the beginning of treatment. ***P<0.001. Error bar, mean±s.e.m. (D) Cyto-ID intensities measured 1 day after DSS treatment shows recovery and non-recovery after single (top panel) and repeated (bottom panel) DSS injury. Altogether for C and D, *N*=544 from six clutches. ***P<0.001. **P<0.01. Error bar, mean±s.e.m. (E) High levels of mortality with *E. coli* treatment following single DSS injury. The left panel plots mortality rates at 1, 2 and 3 h after exposure to heat-killed *E. coli* labeled with Alexa Fluor 488 (green fluorescence) following single DSS injury. *P<0.05, comparing mortality rates at each time point with and without DSS treatment. *N*=341 from three clutches. Error bar, mean±s.e.m. The right panel shows systemic penetration of *E. coli* in the dorsal aorta (upper arrow) and posterior cardinal vein (lower arrow) with DSS treatment imaged 90 min after heat-killed *E. coli* treatment. Scale bars: 20 µm. Full bacterial invasion videos are shown in Movies 6 and 7.

The uptake of bacterial proteins by gut-associated specialized enterocytes and possibly mononuclear phagocytes serves to contain bacterial contaminants to the intestine, a critical protective function. One of the main functions of LREs in zebrafish is endocytosis of proteins from the lumen of the intestine (Rodriguez-Fraticelli et al., 2015). Using fluorescent and confocal microscopy, we sought to visualize the local containment of bacteria in the absence and presence of DSS. We used pHrodo-labeled, heat-killed *Escherichia coli*, which fluoresces at pH levels below 5.5. The amine of *E. coli* proteins was conjugated by activated succinimidyl ester to pHrodo dye. After 1 hour of *E. coli* incubation, we observed markedly increased uptake of *E. coli* proteins with DSS treatment (Fig. 2A). Cyto-ID fluorescently stains autophagosomes in live cells, but does not stain lysosomes (Marx, 2015). To confirm the specificity of Cyto-ID staining for autophagy in the zebrafish intestine, Bafilomycin A1 treatment was combined with both Cyto-ID and Neutral Red staining. Bafilomycin A1 is a late-stage autophagy inhibitor that blocks both starvation-induced and starvation-independent autophagy (Hundeshagen et al., 2011). In the larval zebrafish, inhibiting autophagy leads to loss of Cyto-ID staining but not Neutral Red staining of LREs, indicating depleted autophagy levels but normal levels of functional lysosomes (Fig. S6A,B). Colocalization of labeled *E. coli* proteins and autophagy as measured by Cyto-ID was observed in LREs (Fig. 2B, Movies 1-4). The fluorescence from *E. coli* proteins was most intense in the posterior midgut (Fig. 2A), with pHrodo signals localizing to the region enriched for LREs as shown by LysoSensor-labeled lysosomes (Fig. S7A). Similar to recovery trends seen with Neutral Red staining, we observed markedly decreased Cyto-ID staining with either single or repeated DSS injury (Fig. 2C). The single-DSS-injury finding was further validated with p62 immunoblot (Fig. S8) (Schiebler et al., 2015; Wu et al., 2016). Outside of the posterior midgut, Cyto-ID staining of autophagy is present and not limited to the region with ingested *E. coli* proteins (Fig. S7B). With and without 0.25% DSS treatment, we showed the decrease of Cyto-ID intensity in the LRE region of the posterior midgut (Fig. S9). One course of DSS injury demonstrated a marked recovery of autophagy 1 day after removal of DSS (Fig. 2D); however, similar to trends observed with Neutral Red staining, three courses of DSS injury did not demonstrate recovery of Cyto-ID staining.

**Fig. 3.**
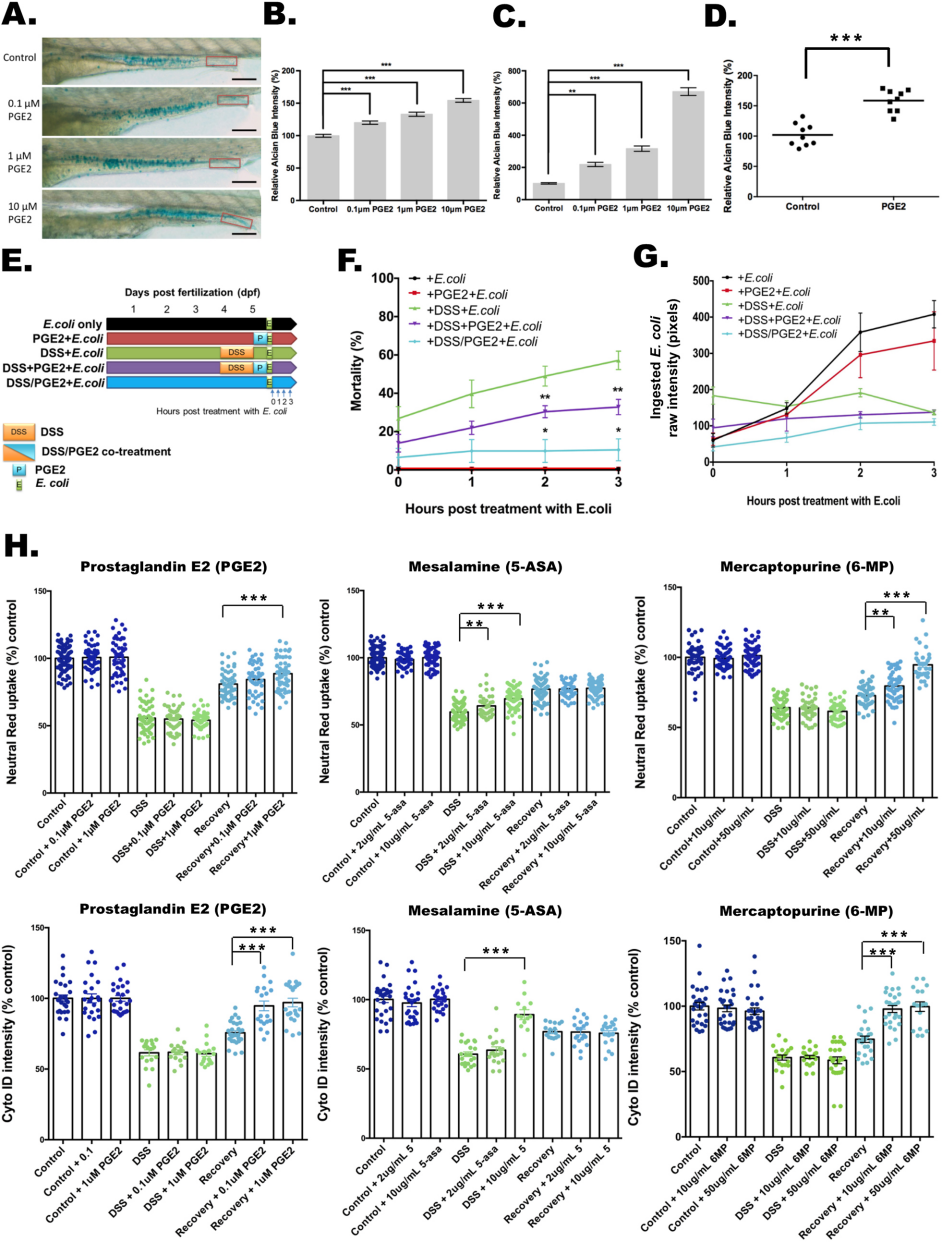
**Effects of PGE2 and commonly utilized IBD medications on barrier function, bacterial containment and LRE function.** (A-C) PGE2 induces mucin expression and release in a dose-dependent manner. (A) The images of Alcian Blue staining with treatments of 0.1, 1 and 10 μM PGE2. The quantification of the whole gut region is shown in B, and the red boxes indicate the quantification areas in C. Scale bars: 100 μm. Alcian Blue intensities of the full intestine (B) and the intestinal lumen (C) are shown (*N*=100 from three clutches). **P<0.01. ***P<0.001. Error bar, mean±s.e.m. (D) Quantification of relative Alcian Blue intensity in a human enteroid differentiated epithelial monolayer with and without 1 μM PGE2 treatment. ***P<0.001. *N*=3 patients with three biopsies each. (E-G) Experiment design of DSS and PGE2 treatment (E) for mortality assays (F) or ingested pHrodo-labeled *E. coli* protein intensity (G). *P<0.05. **P<0.01. *N*=1145 from nine clutches. Error bar, mean±s.e.m. (G) Fluorescent intensity of ingested pHrodo-labeled *E. coli* proteins 1, 2 and 3 h after removal from DSS. *N*=240 from three clutches. Error bar, mean±s.e.m. (H) Applying PGE2 (left panels), mesalamine (middle panels) and 6-mercaptopurine (right panels) to the DSS injury model. The relative Neutral Red (top panels) and Cyto-ID (bottom panels) intensities with treatment alone (dark blue), with treatment during DSS injury for 24 h (light green) and with 5 h of treatment after DSS removal (light blue) are reported relative to untreated controls. **P<0.01. ***P<0.001. Neutral-Red–PGE2, *N*=505 from three clutches. Neutral-Red–mesalamine, *N*=695 from six clutches. Neutral-Red–6-mercaptopurine, *N*=705 from three clutches. Cyto-ID–PGE2, *N*=196 from three clutches. Cyto-ID–mesalamine, *N*=189 from three clutches. Cyto-ID–6-mercaptopurine, *N*=213 from three clutches. Error bar, mean±s.e.m.

Endocytosis of proteins is an essential function of LREs in the zebrafish intestine (Rodriguez-Fraticelli et al., 2015). Plasmolipin (Pllp), a cellular marker for LREs, is also functionally necessary for proper apical endocytosis (Rodriguez-Fraticelli et al., 2015). Taken together, it follows that LREs may have the ability to endocytose the proteins of immotile pathogens. Treatment with high-dose, but not low-dose, DSS followed by *E. coli* exposure results in marked mortality (high-dose: Fig. 2E left panel; low-dose: Fig. S10). Using the zebrafish model, the role of bacterial invasion, an initial step of sepsis and systemic infection, is dramatically demonstrated *in vivo* (Fig. 2E right panel, Movies 6,7) and quantified (Fig. S11). The intensities of fluorescently labeled *E. coli* proteins are high in the intestine (Movies 6,7), which suggests that the initiation of bacterial invasion originates from the intestine. However, further analysis will be needed to exclude the possibility of DSS injury allowing *E. coli* invasion via extra-intestinal surfaces of the zebrafish.

### PGE2 and common IBD medications impact barrier function, bacterial containment and LRE function

We next sought to examine mechanisms modulating the mucus layer, with a particular focus on the effects of treatment with prostaglandin E2 (PGE2). The CD-predominant association to the chromosome 5p13 region containing *PTGER4* is the third most significant association in Europeans, with the *PTGER4* risk alleles correlating with increased expression (Libioulle et al., 2007; Gettler et al., 2019). Contrary to this, however, PGE2 is known to play a critical role in intestinal mucosal cytoprotection mediated by PTGER4 (Takeuchi, 2010). Blockade of PGE2 through the use of COX2 (cyclo-oxygenase 2) inhibitors (e.g. non-steroidal anti-inflammatory agents) impairs mucosal cytoprotection, frequently resulting in intestinal ulceration (Miyoshi et al., 2016). Using our *in vivo* model, we observed a dose-dependent increase in mucus expression by Alcian Blue staining with PGE2 treatment for 24 h (Fig. 3A,B). This increase was even more marked when confining the Alcian Blue quantification to the secreted, luminal mucus staining in the distal hindgut region (Fig. 3C; Oehlers et al., 2012). With DSS injury, we observed the same level of increase of Alcian Blue staining with PGE2 treatment (Fig. S12A). Similar results were obtained with enteroids from human intestinal epithelium, with a marked induction of Alcian Blue staining (Fig. 3D; images: Fig. S12B).

We next sought to define the time-course dependencies of PGE2 treatment relative to DSS injury and bacterial exposure. DSS injury followed by *E. coli* exposure results in high levels of mortality (Figs 2E and 3F, green line). With one course of DSS injury, followed by PGE2, and then *E. coli* treatment (Fig. 3E), we observed a significant decrease in mortality (Fig. 3F, purple compared to green line); concomitant treatment of PGE2 during the DSS injury period, followed by a 5 h recovery period, then *E. coli* treatment, resulted in an even further decrease in mortality (Fig. 3F, blue line). With DSS administration (Fig. 3E-G, green, purple and blue lines), the relative mortality (Fig. 3F) parallels the fraction of ingested *E. coli* proteins at 1 and 2 h post-DSS treatment as measured by pHrodo intensities (Fig. 3G); after 3 h of *E. coli* exposure, among the surviving individuals, all three of the DSS-treated conditions level off to equivalent amounts of ingested pHrodo-labeled *E. coli* proteins. In the absence of DSS treatment (Fig. 3F,G, black and red lines), *E. coli* treatment alone or with antecedent PGE2 treatment before *E. coli* exposure (Fig. 3E, black and red bars), no mortality is detected (Fig. 3F); successively increasing amounts of ingested pHrodo-labeled *E. coli* proteins were observed 1, 2 and 3 h after *E. coli* exposure (Fig. 3G, black and red lines), in amounts that far surpass the DSS-treatment groups (Fig. 3G, green, purple and blue lines). This indicates that, in the absence of DSS treatment, with less abrupt bacterial exposure, robust uptake and containment of bacterial proteins by LREs limits mortality.

Finally, we sought to model the effects of PGE2 as well as drugs commonly utilized in IBD treatment on intestinal LRE phenotypes and function. Administration of either PGE2 or 6-mercaptopurine during DSS injury did not result in enhancement of Neutral Red or Cyto-ID staining compared to DSS treatment alone (Fig. 3H, light-green points). Interestingly, co-treatment of mesalamine during DSS injury resulted in increased Neutral Red staining at both the 13 and 65 µM doses, with an increase in Cyto-ID observed at the 65 µM dose (Fig. 3H, light-green points). Neutral Red and Cyto-ID intensities were higher 5 h after DSS removal (Fig. 3H, light-blue points) compared to measurements taken immediately after DSS removal (Fig. 3H, light-green points). Treatment with mesalamine administered for 5 h immediately after removal of DSS (Fig. 3H, light-blue points, recovery phase) did not result in enhancement of Neutral Red or Cyto-ID intensities compared to untreated recovery controls. In contrast, 5 h of PGE2 treatment in the recovery phase resulted in significant induction of Neutral Red and Cyto-ID intensities (Fig. 3H, light-blue points), presumably reflecting the mucus cytoprotective effects of PGE2 (Fig. 3A-D). Importantly, 6-mercaptopurine treatment resulted in increased Neutral Red and Cyto-ID intensities at both the 66 and 330 µM doses (Fig. 3H, light-blue points), demonstrating a surprisingly rapid *in vivo* induction of autophagy in the recovery period following DSS injury.

## DISCUSSION

Traditionally, the most commonly utilized models in IBD involve murine models, including IL10-pathway knockouts, the T-cell transfer model, DSS treatment and, more recently, the *abcb1a* knockout (*mdr1a^−/−^*), which accurately predicted human responses to blockade of IL12/23 and IL17 as being beneficial and harmful, respectively (Keubler et al., 2015; Ostanin et al., 2009; Chassaing et al., 2014; Wilk et al., 2005). However, long-term remission rates, as measured by mucosal healing at week 4, indicated that development of agents that address the epithelial alterations in IBD are needed (Holleran et al., 2017). In particular, given the known role that decreased autophagy in epithelial cells plays in driving CD (Ke et al., 2016; Iida et al., 2017), development of *in vivo* models that can rapidly scale testing of new agents is needed.

Zebrafish models of IBD provide substantive advantages in modeling repetitive intestinal injury and repair. In this regard, the most broadly utilized models of IBD have used DSS, which results in epithelial injury and inflammation, followed by prompt recovery following DSS removal. It has been reported that DSS injury results from complex formation with intraluminal fatty acids during DSS administration, resulting in impaired barrier function. Chronic models of DSS in mice have been reported, but take months to develop (Wirtz et al., 2017). In the present models, we observe a striking failure to fully recover from repetitive DSS injury as manifested by increased mortality, impaired mucus production and impaired autophagy, all within 2 weeks; although the inflammatory response is predominant in the intestine, given the mode of administration, extraintestinal effects may occur. We observed the incomplete recovery with repetitive injury both by measurements of Neutral Red, which measures lysosomal pH, as well as by CytoID, which measures autophagosomes.

**Fig. 4.**
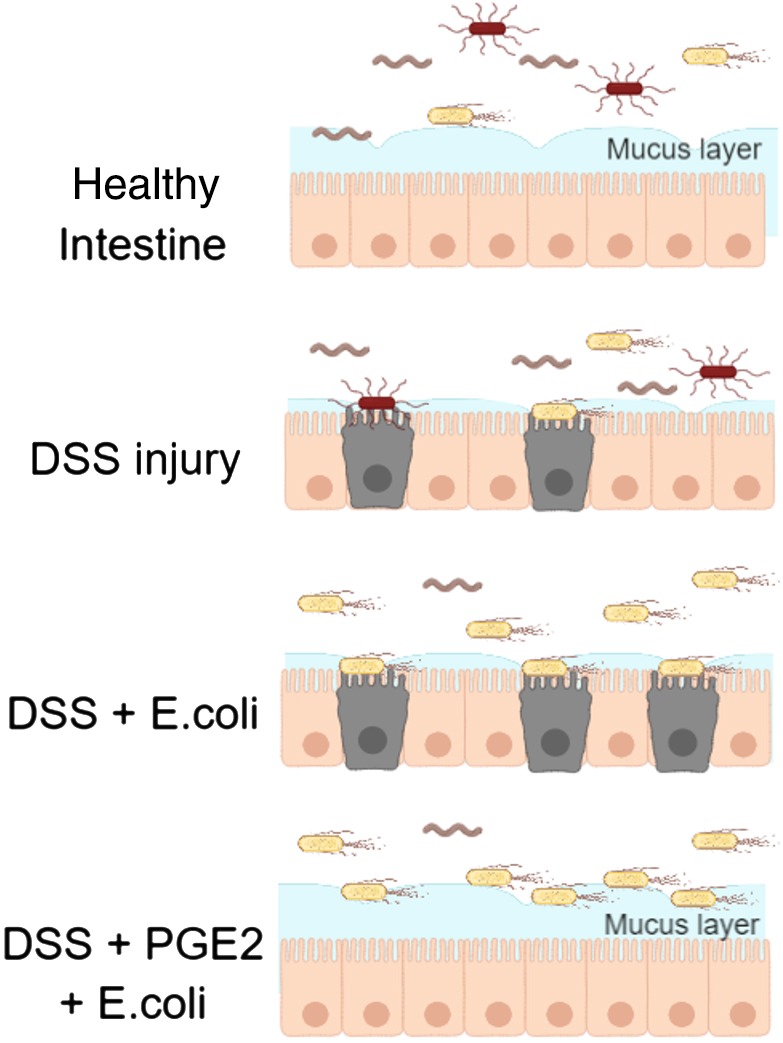
**Effects of PGE2 on mucus production and barrier protection.** In healthy intestine, the mucus layer prevents microbes/microbial products from making contact with the epithelial barrier. With DSS injury, the mucus is depleted and the epithelial barrier is damaged, allowing microbes/microbial products access to the barrier. *Escherichia coli* incubation following DSS treatment yields increased *E. coli* in the intestine and increased bacterial contact with the epithelial cells. PGE2 rescues loss of the mucus layer due to DSS injury, preventing *E. coli* from reaching the epithelial barrier.

The high mortality observed with repetitive DSS injury was associated with a striking, systemic presence of microbial products (Fig. 2F). Both *in vivo* and *in vitro* (Fig. 3A-G), we observed that PGE2 potently induces mucus expression, which has previously been reported to be mediated by prostaglandin E2 receptor EP4 subtype (PTGER4)-mediated mucin exocytosis in a human carcinoma cell line (Belley and Chadee, 1999). Furthermore, mice deficient in PTGER4 in the intestinal epithelial layer did not develop wound-associated epithelial cells, resulting in impaired tissue repair (Miyoshi et al., 2016). Our data highlight the critical role that PGE2 plays in protecting against intestinal-barrier-associated bacterial invasion through increased mucin production, blocking uptake of luminal *E. coli* proteins (Fig. 4). These findings are consistent with a critical role of the PGE2-PTGER4 pathway in mediating intestinal barrier function, and administration of PGE2 after removal of DSS resulted in enhanced recovery of Cyto-ID staining (Fig. 3H). The complex actions of PGE2-PTGER4 in intestinal barrier function, epithelial repair and innate immune functions have yet to be fully elucidated. The presence of CD-predominant *PTGER4* associations that increase *PTGER4* gene expression (Libioulle et al., 2007) would appear to contradict the overall protective actions of PGE2-PTGER4 in intestinal barrier and epithelial barrier function. Future studies evaluating pathogenic effects of increased *PTGER4* expression, especially in CD, are of the highest priority.

During DSS injury, only mesalamine resulted in enhanced Cyto-ID staining; this finding is consistent with prior literature that mesalamine acts by blocking pro-inflammatory-cytokine-mediated induction of NF-κB, thereby enhancing epithelial barrier function (Kaiser et al., 1999; Di Paolo et al., 1996). In our study, mesalamine exerts its protective effects only during injury and not during the recovery period following DSS removal. In contrast, 6-mercaptopurine enhances autophagy solely after DSS removal, consistent with prior literature of direct effects of thiopurines in enhancing autophagy (Chaabane and Appell, 2016). While blockade of pro-inflammatory cytokines such as anti-TNF or anti-IL12/23 (Sandborn et al., 2012) represents major mechanisms for treating moderate to severe CD, it is still an open question as to whether management combining pro-inflammatory cytokine blockade (Abraham et al., 2017) with 6-mercaptopurine provides additional benefit (Colombel et al., 2010). As with many highly utilized, older medications, the precise mechanism of action of 6-mercaptopurine is incompletely understood and likely multifactorial in nature (Ordás et al., 2012; Poppe et al., 2006). Its use has been supplanted by more potent pro-inflammatory blockade in moderate to severe disease. While the CD-predominant associations to autophagy represent some of the highest effect and previously unappreciated pathways implicated by genome-wide association studies, given the overall lower efficacy of 6-mercaptopurine in moderate to severe CD (Colombel et al., 2010), our studies provide a cautionary note regarding the potency of CD-based therapeutic development focused solely on enhancing autophagy in the intestine. In the era of precision medicine, more rapid scaling of a sophisticated understanding of time course factors, disparate cellular effects and mechanisms of action of established and new drugs will be required. While mammalian systems will remain a linchpin, the superior speed, sample size, visualization (e.g. microbes, autophagy) and key role of the epithelial barrier makes high-throughput studies in the zebrafish an important component of the IBD therapeutic development pipeline.

## MATERIALS AND METHODS

### Zebrafish maintenance

Adult zebrafish were maintained on a 14:10 h light:dark cycle at 28°C. Wild-type (WT; AB and Tab 14) and *Tg(mfap4:turquoise^xt27^)* (Oehlers et al., 2015; Walton et al., 2015) fish were used. Fertilized embryos collected following natural spawning were cultured at 28.5°C in egg water (0.6 g/l Instant Ocean; Blacksburg, VA, USA) containing Methylene Blue (0.002 g/l) and 75 μM 1-phenyl 2-thiourea. The Mount Sinai School of Medicine Institutional Animal Care and Use Committee approved all protocols. For fluorescent imaging, Methylene Blue was removed within 24 h of embryo collection. Larvae and adults were fed once a day with Hikari First Bites (Hikari) at 6 days post-fertilization and Zeigler Zebrafish Diet with Hatching Brine Shrimp Eggs (Pentair Aquatic Eco-Systems, FL, USA), respectively.

### Enteroid culture establishment and differentiation

Human biopsy collection was approved by the Institutional Review Board of the Mount Sinai School of Medicine. Human enteroid lines were generated according to the method from VanDussen et al. (2015) with modifications. Briefly, biopsies were obtained from healthy and IBD individuals during routine colonoscopy visit. Biopsies were dissociated by collagenase I (Thermo Fisher Scientific) incubation at 37°C for 30 min and vigorous pipetting of mixtures every 5 min. Biopsy mixtures were passed through a 70-µm cell strainer (BD Biosciences) followed by two washes with wash medium (DMEM/F12 medium with 15 mM HEPES, 2 mM L-glutamine, 100 U/ml of penicillin and 0.1 mg/ml of streptomycin). The crypt mixtures were suspended in Matrigel (Corning) and cultured in 50% L-WRN (L-cell expressing Wnt3a, Rspondin3 and noggin) conditioned medium with 10 µM of Y-27632 (ROCK inhibitor; R&D Systems) and 10 µM SB-431542 (TGF-βR inhibitor; R&D Systems). L-WRN conditioned media was prepared as described (Miyoshi and Stappenbeck, 2013). Cells were passaged every 4-6 days by trypsinization, and fresh 50% L-WRN was replaced every 2 days. After five passages, cells were frozen in 8.3% DMSO in wash media and kept in liquid nitrogen. Prior to differentiation, cells were thawed and expanded in 50% L-WRN with 10 µM of Y-27632 and 10 µM SB-43154. To induce differentiation, 2.5×10^5^ of trypsinized spheroids were seeded in 6.5-mm PET Transwell inserts (Corning) precoated with 1% gelatin and cultured in 5% L-WRN with 10 µM of Y-27632 for 3 days. For mucus quantification studies, 1 µM of PGE2 or the same volume of ethanol control was supplement in 5% L-WRN during the last 24 h of the differentiation.

### Single and repeated DSS injury model

The single-injury protocol was adapted and modified from Oehlers et al. (2013). Batches of 60 larvae were segregated into Petri dishes in 50 ml of egg water. Phenylthiourea (75 μM; Acros Organics) was added 24 h post-fertilization to prevent pigment-cell formation. To induce intestinal injury, 3 days post-fertilization (dpf) larvae were placed in freshly prepared 0.1 or 0.25% (w/v) colitis grade DSS (36,000-50,000 MW, MP Biomedical) for 3 days. In the repeated-injury protocol, larvae were treated with 0.05, 0.1 or 0.25% DSS at 5, 8 and 11 dpf for 24 h, followed by removal of DSS. Larvae were not fed for the duration of the single DSS treatment experiments (6 dpf); the larvae were fed after 7 dpf with repeated DSS. Feeding was skipped on the DSS treatment day.

### Automated hematoxylin and eosin (H&E) staining

An Autostainer XL (ST5010, Leica Biosystems, Nussloch, Germany) was used for H&E staining. The automated staining protocol consisted of the following steps: three changes of xylenes (2, 2 and 1 min), rehydration in an ethanol/water gradient (100%, 100%, 95% ethanol for 2, 1 min and 20 s, respectively), followed by washing with deionized (DI) water (2 min). The rehydrated slides were stained with Gill 3 Hematoxylin (12 min; Sigma-Aldrich), washed with DI water (2 min), followed by 0.17% acid alcohol (1 min), washed with DI water (1 min), ammonia water (20 s), washed with DI water (1 min), and 95% ethanol (10 s). Staining with eosin (45 s; Sigma-Aldrich) was completed, followed by four washes with 100% ethanol (1 min each), and four washes with xylene (1 min each). H&E-stained slides were coverslipped with Cytoseal XYL (Thermo Fisher Scientific) and dried prior to imaging.

### Neutral Red and Alcian Blue staining and image quantification

The Neutral Red and Alcian Blue staining protocols were adapted and optimized from Oehlers et al. (2013). Zebrafish larvae were stained live with 2.5 μg/ml Neutral Red (ACROS, Bridgewater, NJ, USA) in egg water for 5 h. The intensity of Neutral Red staining is non-saturated and consistent between 4 and 6 h (Fig. S2A). After moving stained larvae to fresh 50 ml egg water, larvae were anesthetized in Tricaine-S (Western Chemical), followed immediately by live imaging. For the zebrafish intestinal injury model, mucin was stained as the quantifiable metric. Larvae were fixed in 4% paraformaldehyde at room temperature for 2 h, rinsed twice with acid alcohol (70% ethanol with 1% hydrochloric acid) and stained in 0.01% Alcian Blue (w/v, in 80% ethanol with 20% glacial acetic acid) for 3 hours at room temperature. The Alcian Blue intensities were linear and non-saturated at 2-4 h of staining (Fig. S4A,B). After rinsing with acid alcohol three times, fixed larvae were imaged on a thin layer of 5% agarose gel.

For Alcian Blue staining of differentiated human enteroids, cells were fixed in cold methanol-Carnoy solution (60% methanol, 30% dry chloroform, 10% glacial acetic acid; Sigma) overnight and washed with 1× phosphate-buffered saline (PBS; 10 mM PO_4_^3−^, 137 mM NaCl and 2.7 mM KCl) three times. Then, cells were kept in 1× PBS with 0.02% acetic acid solution for an additional 16 h at 4°C. Prior to Alcian Blue staining, cells were kept in 3% acetic acid for 3 min at room temperature. Cells were stained with 1% Alcian Blue (in 3% acetic acid solution; ACROS Organics) for 30 min at room temperature followed by three washes in 1× PBS solution. Transwell membranes were cut and mounted in fluoromount G (Electron Microscopy Sciences) and visualized.

For both Neutral Red and Alcian Blue staining, the images of full gut and/or lumen were collected under 100× total magnification with the EVOS XL core microscope (Thermo Fisher Scientific). For Alcian Blue images, we extracted intensity from the blue channel before we performed quantification. For both Neutral Red and Alcian Blue staining, we inverted the color of the image, traced the gut and/or lumen, or monolayer cells from differentiated enteroids, and measured the mean intensity per pixel using ImageJ software. To remove background signals, we subtracted the mean intensity per pixel of images from the anal end of the gut. The relative expressions (%) were calculated by normalizing to the untreated group. Statistical tests were done with two-tailed Wilcoxon-test in RStudio.

### Bacterial infection and autophagy

Heat-killed *E. coli* (K-12 strain) were conjugated either with BioParticle–Alexa-Fluor-488 conjugates or pHrodo Red *E. coli* BioParticles Conjugate for uptake studies (ThermoFisher, Waltham, MA, USA). Bacterial concentrations were titrated and optimized based on intestinal fluorescent intensity. Zebrafish larvae were incubated in 2 μg/μl (6×10^7^ CFU/ml) for 1 hour. Larvae were rinsed and moved to egg water, with mortality and fluorescence measured at 0, 1, 2 and 3 h.

We used a Cyto-ID Autophagy detection kit 2.0 (Enzo Biochem) to analyze autophagy with DSS and/or bacterial infection in live zebrafish. Zebrafish were incubated in 1 μl CYTO-ID per 500 μl egg water for 1 hour with or without *E. coli* or DSS and washed with 50 ml egg water before imaging. The fluorescent images for quantification were collected after *E. coli* treatment with the EVOS FL Cell Imaging System. Using ImageJ software, we measured the mean intensity per pixel for the gut and subtracted this background for individual images. Auto-fluorescence was corrected by comparing to the unstained fish gut. The relative expressions (%) were calculated by normalizing to the untreated group. Statistical tests were done with Wilcoxon-test in RStudio.

The confocal images of colocalization analysis and movies were generated with the ZEISS LSM 880 with Airyscan. For colocalization, *Tg(mfap4:turquoise)* fish were treated with pHrodo Red *E. coli* and stained for autophagy with CytoID. The confocal *z*-stack movies (Movies 1-4) and cell migration movies (Movie 5) were collected after 5 h of *E. coli* treatment. Systemic infection movies (Movies 6,7) were imaged with BioParticle–Alexa-Fluor-488 conjugate at 90 min after the end of *E. coli* treatment.

### PGE2 and drugs commonly utilized in IBD treatment

PGE2 and IBD drugs were added directly to zebrafish media either with or without DSS at 5 dpf for co-treatment as a therapy. In the recovery groups, drugs were added after removing DSS, and imaging was performed 5 h later. The doses of drugs were tested and the highest doses without fish death were chosen. Final concentrations of PGE2 were 0.1 and 1 µM with equal amounts of ethanol used for controls. Mesalamine (5-aminosalicyclic acid) was dissolved in egg water at a concentration of 10 mg/ml and adjusted to pH 7 with dilute sodium hydroxide. 6-Mercaptopurine was at a concentration of 35 mg/ml dissolved and diluted to the final concentrations with egg water, with pH adjusted to 7.

### Gene expression analysis of zebrafish intestines

From anesthetized 6-dpf zebrafish larvae, whole intestines were microdissected and separated from the remaining carcasses, with each collected in 30 µl RLT Buffer (Qiagen). Total RNA was isolated from *N*=15-25 intestines or carcasses per sample by TRIzol extraction (Life Technologies). cDNA libraries were generated by reverse transcription using the SuperScript cDNA synthesis kit (Quanta). Quantitative real-time PCR (qRT-PCR) was performed using PerfeCTa SYBR Green Fast Mix (Quanta) with the LightCycler 480 (Roche). Gene expression levels were normalized to ribosomal protein large P0 (*rppo*) using the comparative threshold cycle (ΔΔCt) method. Primer sequences are listed in Table S1.

### Single-cell suspension of zebrafish intestines for flow cytometry

At 6 dpf, zebrafish were stained with LysoTracker Green DND-26 (Invitrogen) at a 1:100 dilution for 1 h. Whole intestines were then dissected from anesthetized zebrafish (*N*=25 per group) and collected in 1 ml MACS Rinsing Solution (Miltenyi Biotech) with 0.5% BSA (Fisher Scientific), followed by the single-cell suspension protocol (Bresciani et al., 2018). In short, samples were rocked at room temperature for 30 min before being centrifuged at 700 ***g*** for 5 min. The cell pellet was suspended in PBS with 1 mg/ml DNase I (Sigma Aldrich) and 1 mg/ml collagenase IV (Sigma Aldrich), followed by harsh pipetting and incubation at room temperature for 5 min. Samples were centrifuged at 700 ***g*** for 5 min and the supernatant was removed. The cell pellet was resuspended in DPBS 1× with 0.5% BSA (Gibco) before being strained through a 70-µm mesh. Single-cell suspensions were flow sorted in the Mount Sinai Flow Cytometry CoRE using the BD influx cell sorter (BD Biosciences). RNA was extracted using the RNAqueous kit (Invitrogen). cDNA synthesis and qPCR were conducted as described in the Gene Expression Analysis section.

### Immunoblot analyses

At 6 dpf, whole zebrafish larvae were homogenized in lysis buffer [20 mM Tris pH 7.5, 150 mM NaCl, 1% NP-40, 2 mM EDTA, 10% glycerol, 1% BME, and protease inhibitors (Roche Complete)]. From each sample, 10 µg of protein was separated on a Mini-Protean TGX 4-15% (Bio-Rad) gel and transferred to a PVDF membrane using a Bio-Rad electrophoretic transfer apparatus (Trans-Blot Turbo, Bio-Rad). After blocking with 5% BSA, the membrane was probed with the primary antibodies anti-p62 (PM045, polyclonal, MBL; 1:1000) and anti-tubulin (12G10, DSHB; 1:2000). A secondary incubation of either 1:40,000 dilution of horseradish peroxidase (HRP)-conjugated goat anti-mouse or 1:20,000 dilution goat anti-rabbit secondary antibody was applied to the membrane. The immunoblots were developed with SuperSignal West Pico (Life Technologies).

### Colocalization analysis of LysoSensor and Neutral Red

The confocal images (Fig. S2B, top panel) of zebrafish intestine stained with LysoSensor and Neutral Red were taken with a Zeiss LSM 880 confocal microscope using 20× air lens (NA=0.8). The zoomed-in images (Fig. S2B, bottom panel) were generated from the non-saturated area in intestine of the original images using Zen 2.3 Lite (Zeiss). Colocalization analysis of both LysoSensor- and Neutral-Red-positive cells was performed using ImageJ Fiji software. The 2D intensity histogram and Pearson correlation coefficient were generated by the Coloc2 plugin of Fiji.

### Quantitation of *E. coli* intensity in cells of zebrafish dorsal aorta and cardinal vein

Zebrafish were treated in the presence or absence of 0.25% DSS for 24 h before being subjected to Alexa-Fluor-488-labeled heat-killed *E. coli* incubation (for 60 min). Movies were taken at 90 min post *E. coli* incubation. The *E. coli* fluorescence intensity of 170 of zebrafish cells were quantified using Zen 2.3 Lite software from Zeiss (38 cells for control-aorta: control treatment in dorsal aorta; 53 cells for DSS-aorta: DSS treatment in dorsal aorta; 38 cells for control-vein: control treatment in posterior cardinal vein; 41 cells for DSS-vein: DSS treatment in posterior cardinal vein). *P*-values were tested by the Mann–Whitney test.

### Chemical inhibitor for autophagy

Bafilomycin A1 (Sigma Aldrich) was dissolved in DMSO to create a 100 μM stock solution and then diluted to a concentration of 10 and 20 nM in egg water. Zebrafish larvae at 5 dpf were treated for 24 h, followed by Cyto-ID and Neutral Red staining.

## Supplementary Material

Supplementary information
