# Supplementary material for: Zebrafish modeling of intestinal injury, bacterial exposures and medications defines epithelial in vivo responses relevant to human inflammatory bowel disease
Source: Dis Model Mech. 2019 Aug 13;12(8):dmm037432. doi: 10.1242/dmm.037432 (PMC6737949; doi:10.1242/dmm.037432)
Supplement: Supplementary information [file dmm-12-037432-s1.pdf]

Supplementary Figures

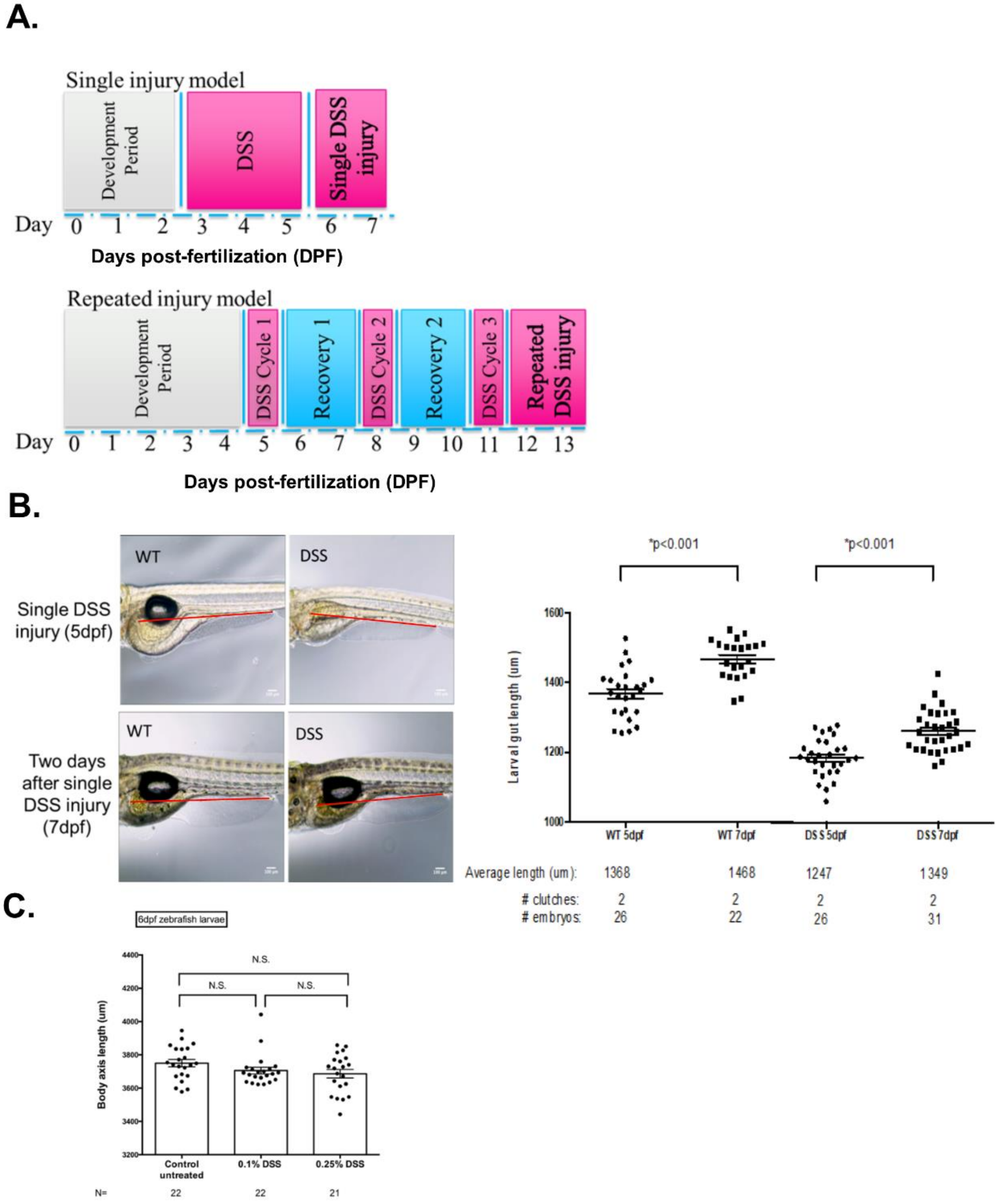

Fig. S1 Gut length is shortened with DSS injury. (**A**) Timeline of single DSS and repeated DSS injuries. (**B**) Zebrafish images of untreated and single DSS injury (left panel). Red lines indicate the measurement of gut length. Quantification of gut length (right panel). Comparing to untreated controls, the gut length is significantly shortened after single DSS injury (P<0.001, N= 149 from 7 clutches). (**C**) The body axis length of untreated and 0.1% and 0.25% DSS treated larvae.

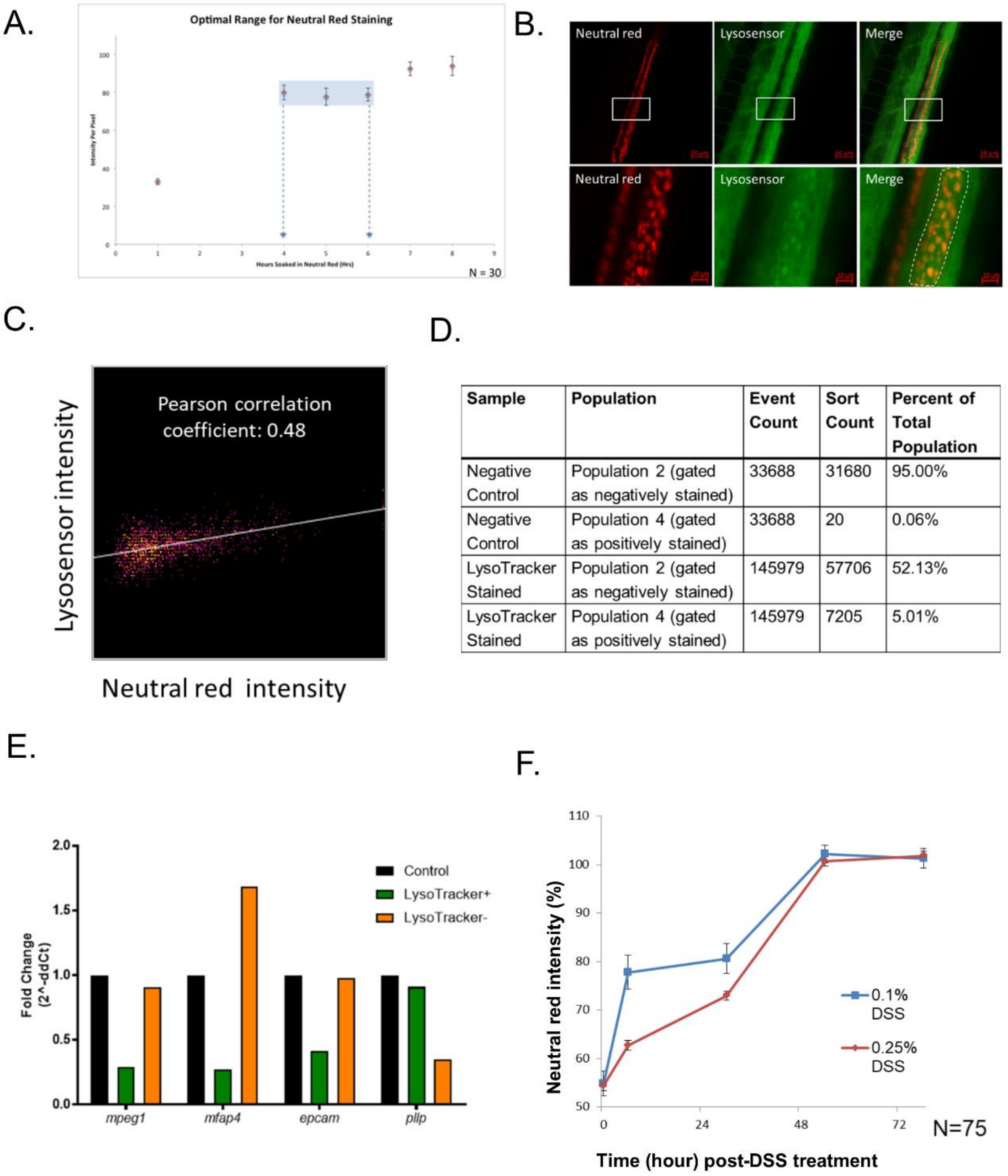

Sample	Population	Event Count	Sort Count	Percent of Total Population
Negative Control	Population 2 (gated as negatively stained)	33688	31680	95.00%
Negative Control	Population 4 (gated as positively stained)	33688	20	0.06%
LysoTracker Stained	Population 2 (gated as negatively stained)	145979	57706	52.13%
LysoTracker Stained	Population 4 (gated as positively stained)	145979	7205	5.01%

Fig. S2. Optimization of neutral red staining. (**A**) Calibration curve of neutral red staining. Neutral red staining equilibrium is between 4-6 hours. N=30 (**B**) Images of Neutral red co-localizes with the lysosomal marker Lysosensor in zebrafish intestine (top panel). Total magnification 200X. Bar, 50µm. Zoom in quantification images (bottom panel) from selected area (white rectangle on top panel). Bar, 10µm. (**C**) Pearson correlation coefficient of Lyso-sensor and Neutral Red intensities in (B) bottom panel. (**D-E**) Lysosome-rich enterocyte (LRE) marker pllp enriched in Lysotracker-positive cells. 15 larvae intestines were dissected and cells were collected after single cell isolation with both Lysotracker staining and no staining control. The sorting result (**D**) showed 5.01% Lysotracker positive cells with minimum contamination (less than 0.06%). (**E**) qPCR results of marker for macrophages (mpeg1), mfap4, epithelia

Disease Models & Mechanisms • Supplementary information

(epcam) and LREs (pllp) in Lysotracker positive, negative relative to unsorted control cells. (**F**) The intestinal neutral red intensity recovers by 54 hours after removal from single DSS injury (N=75 from 3 clutches).

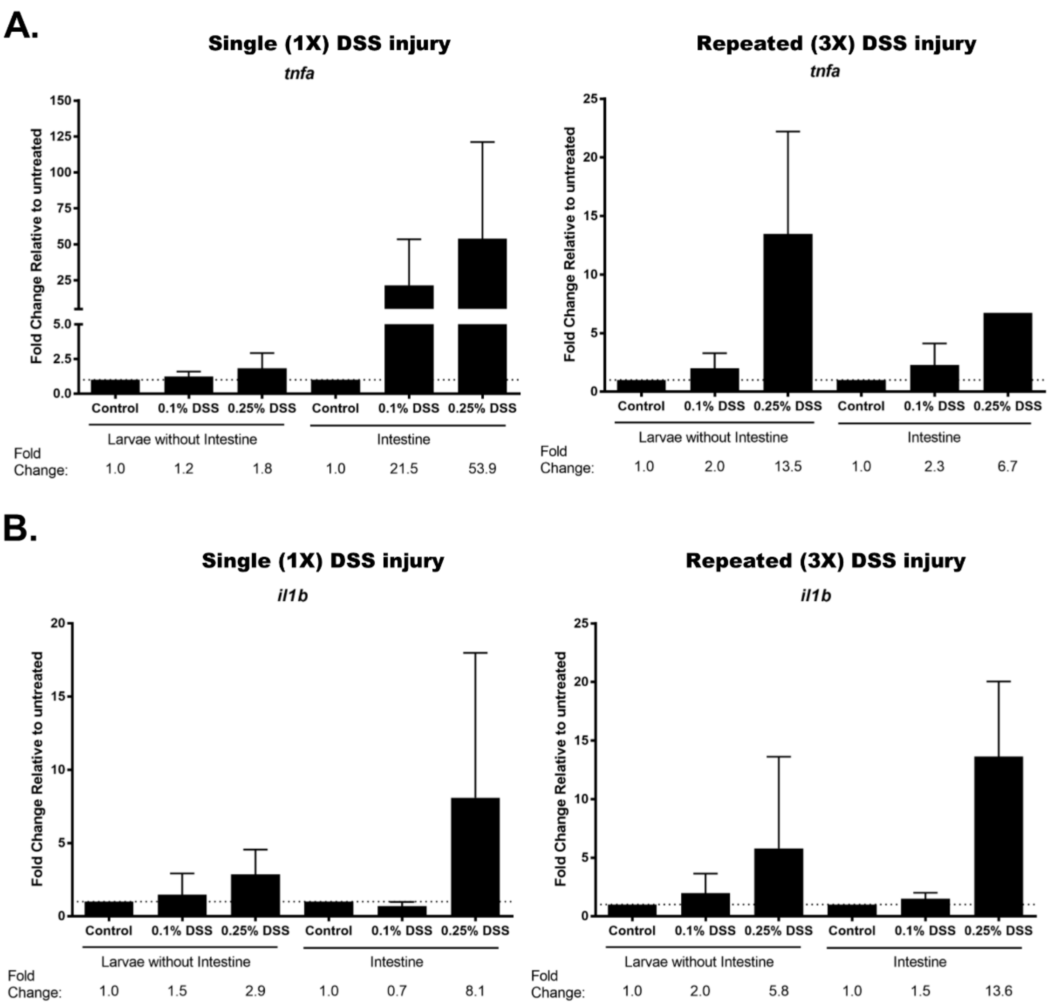

Fig. S3. Inflammatory cytokines TNFα and IL-1b mRNA are predominately induced in intestine with single DSS injury but are also expressed in non-intestinal tissues after repeated injury. (**A**) TNFα expression after single (left) and repeated (right) injury (**B**) IL-1β expression after single (left) and repeated (right) injury.

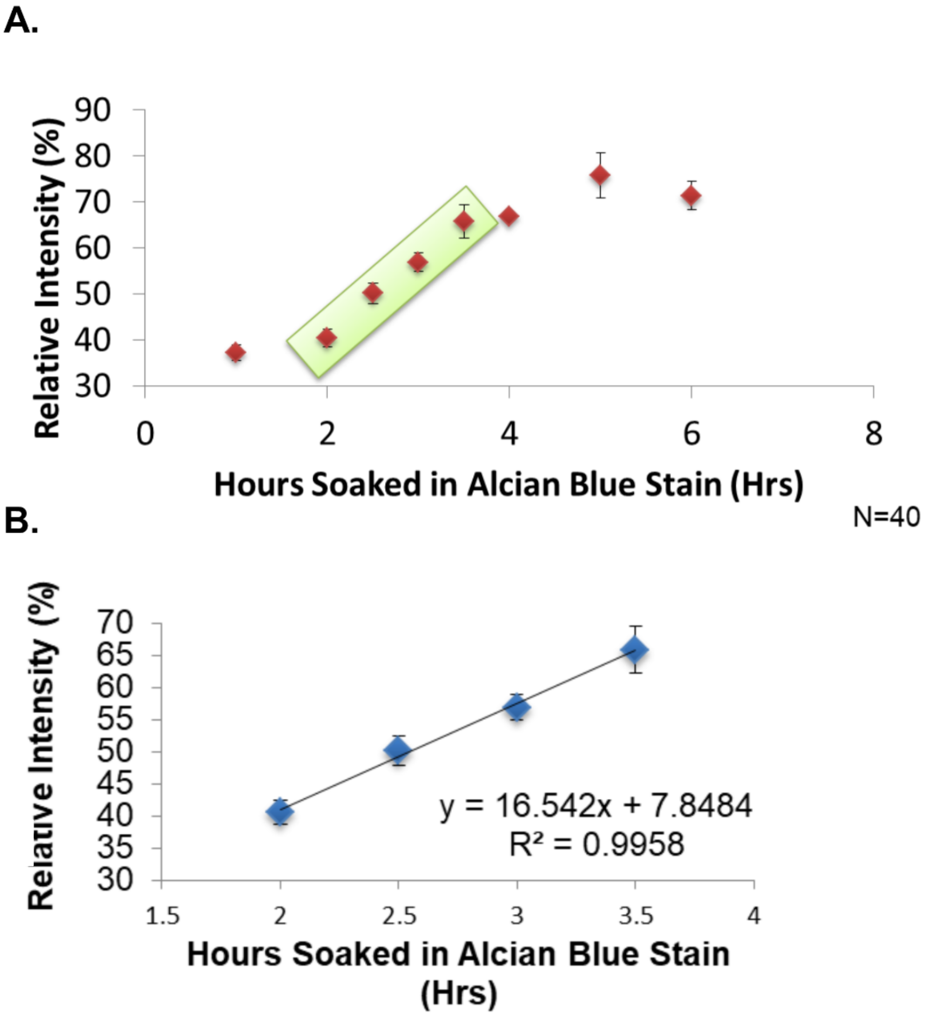

Fig. S4. Optimization of Alcian blue staining. (**A**) Calibration curve of Alcian blue staining. The intensity of Alcian blue staining is linear but not saturated between 2 - 3.5 hours (N=40 from 3 clutches). (**B**) The R^2 is 0.996 for the linear range.

Disease Models & Mechanisms • Supplementary information

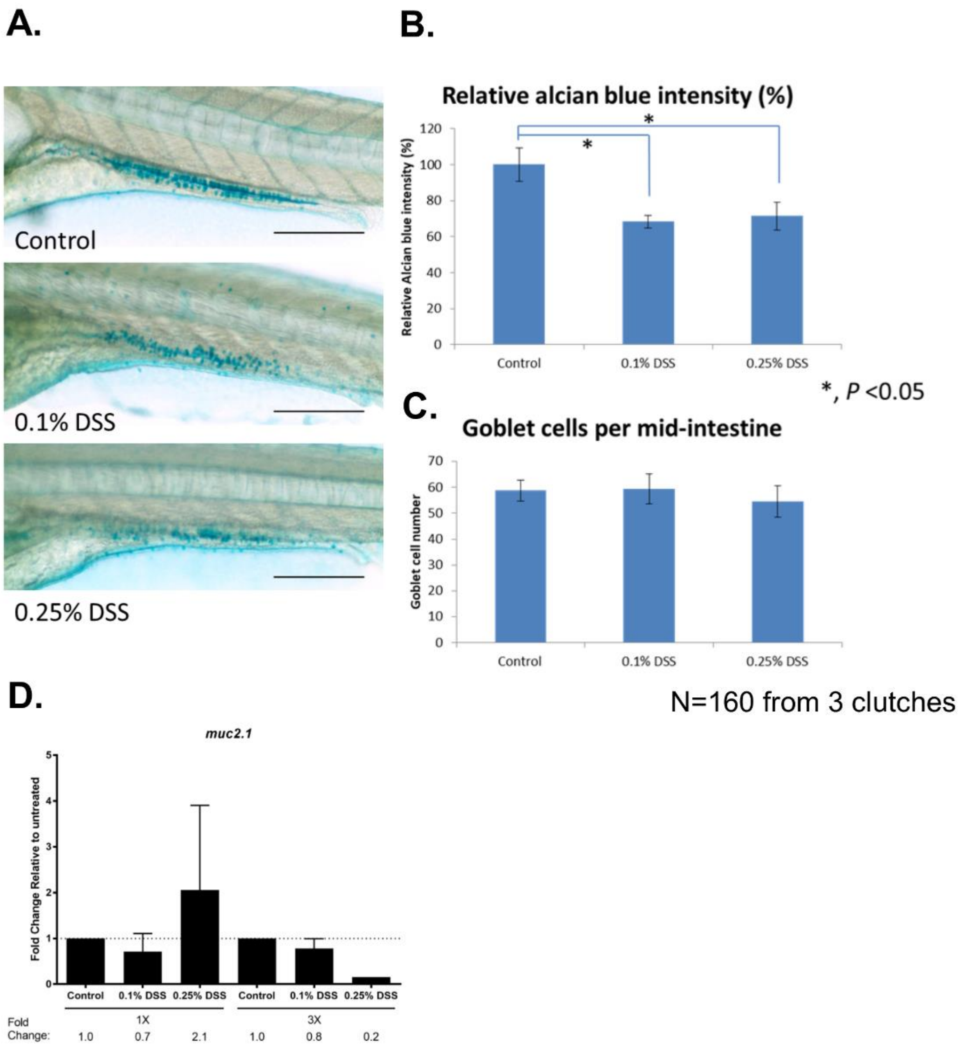

Fig. S5. DSS reduces Alcian blue intensity after single DSS injury but does not affect goblet cell numbers. (**A**) Images of Alcian blue staining with 0.1% and 0.25% DSS treatment. The data was collected at the same time point as 0 hour in Figure 3e right panel. (**B**) Quantification of Alcian blue intensity relative to control. (**C**) Quantification of goblet cells per mid-intestine. N=160 from three clutches. (**D**) qPCR results of muc2.1 with single and repeated DSS injuries.

Disease Models & Mechanisms • Supplementary information

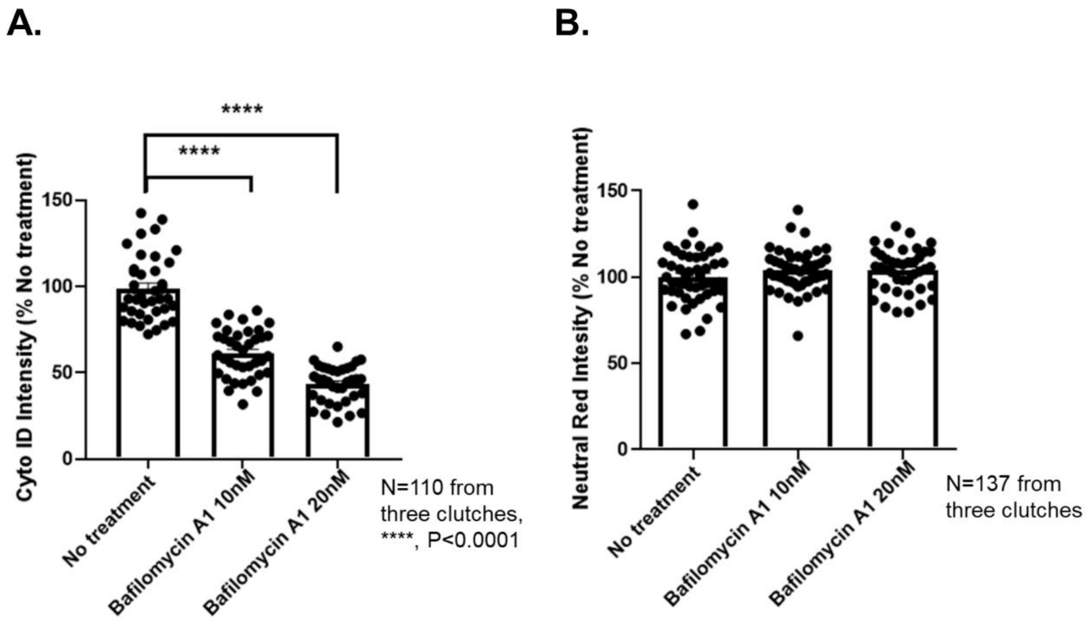

Fig. S6. Treatment of selective autophagy inhibitor Bafilomycin A1. (**a**) Dose-dependent decrease of Cyto-ID staining with 10 and 20nM Bafilomycin A1 after 24 hours of treatment. ****, P<0.0001 (**b**) No change in Neutral Red intensity.

Disease Models & Mechanisms • Supplementary information

A.

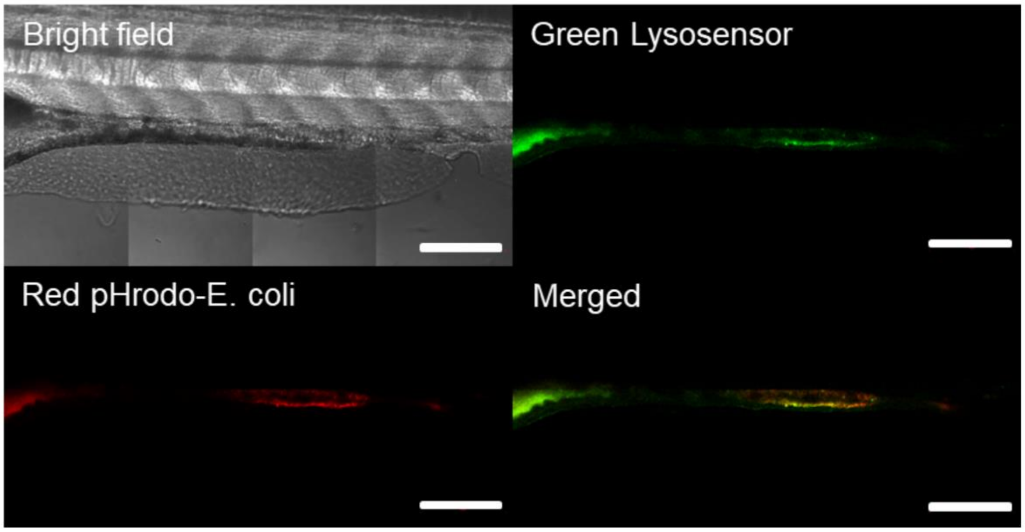

B.

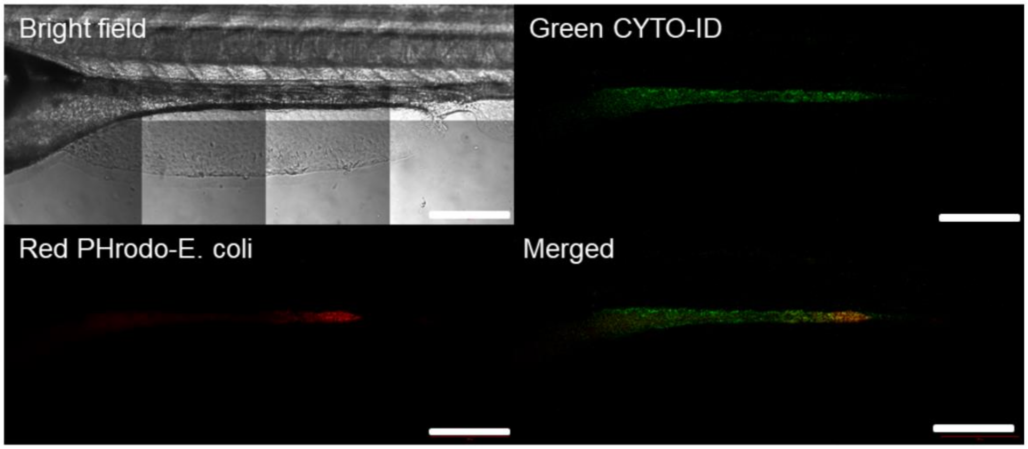

Fig. S7. Confocal tiled image with the full zebrafish larvae intestine. (**a**) The images of bright-field, lysosensor (green), pHrodo-E.coli (red) and merged images of red and green. pHrodo-labelled E. coli were ingested in LRE-enriched region of the intestine. Bar, 200 µm. (**b**) The images of bright-field, Cyto-ID (green), pHrodo-E.coli (red) and merged images of red and green. Cyto-ID staining is present and not limited to the region with ingested E. coli. Bar, 200 µm.

Disease Models & Mechanisms • Supplementary information

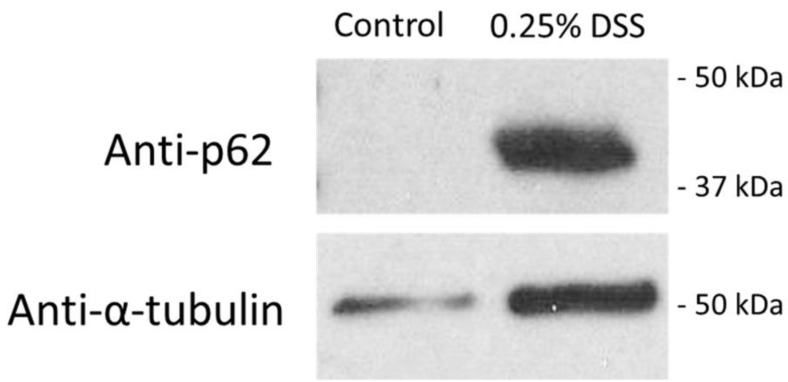

Fig. S8. Immunoblot of the autophagy marker p62 with and without one course 0.25% DSS treatment. p62 proteins accumulate in response of loss of autophagosomes [1, 2]. In Agreement with CytoID results (Fig. 2C), 0.25% DSS treatment induces accumulation of p62.

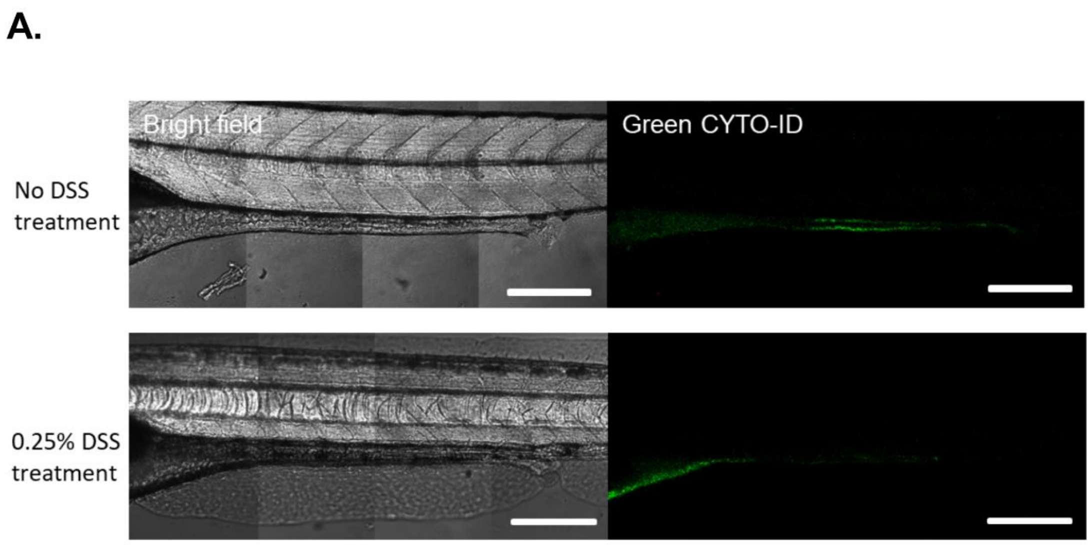

Fig. S9. Confocal tiled image with the full zebrafish larvae intestine with and without DSS treatment. (**a**) The images of bright-field and Cyto-ID (green). Bar, 200 µm.

Disease Models & Mechanisms • Supplementary information

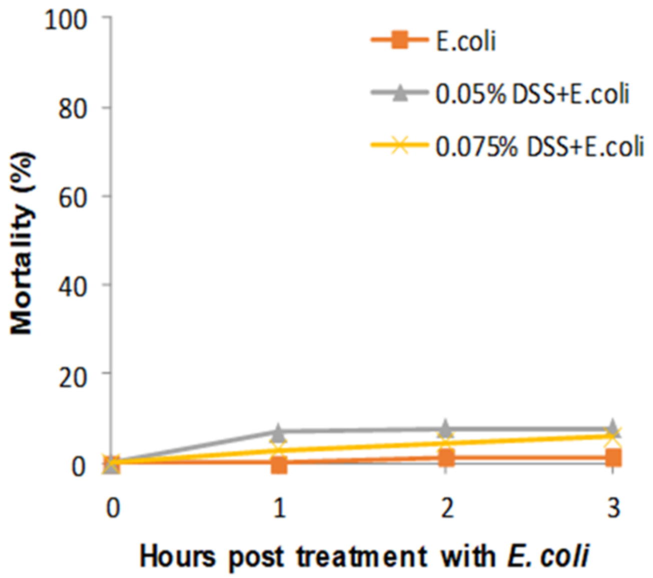

Fig. S10. Low levels of mortality with *E .coli* treatment following single low dose DSS injury. The mortality rates after 1, 2 and 3 hours of heat-killed *E. coli* exposure following single DSS injury. N=241.

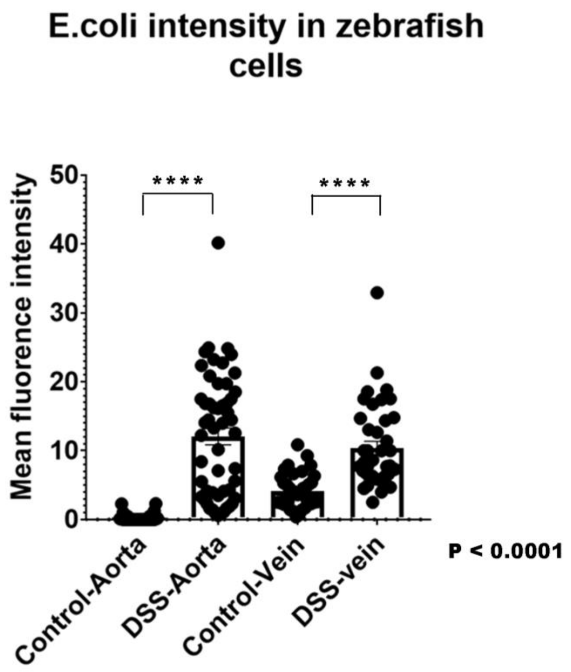

Fig. S11. Quantification *E. coli* intensity in zebrafish aorta and vein with and without DSS treatment. The *E.coli* fluorescence intensity of total 170 of zebrafish cells from two replicated experiments were quantified (See Supplementary Methods). With DSS treatment, there are significantly higher levels of *E.coli* uptake as measured by mean fluorescence in both dorsal aorta and posterior cardinal vein. ****, P< 0.0001.

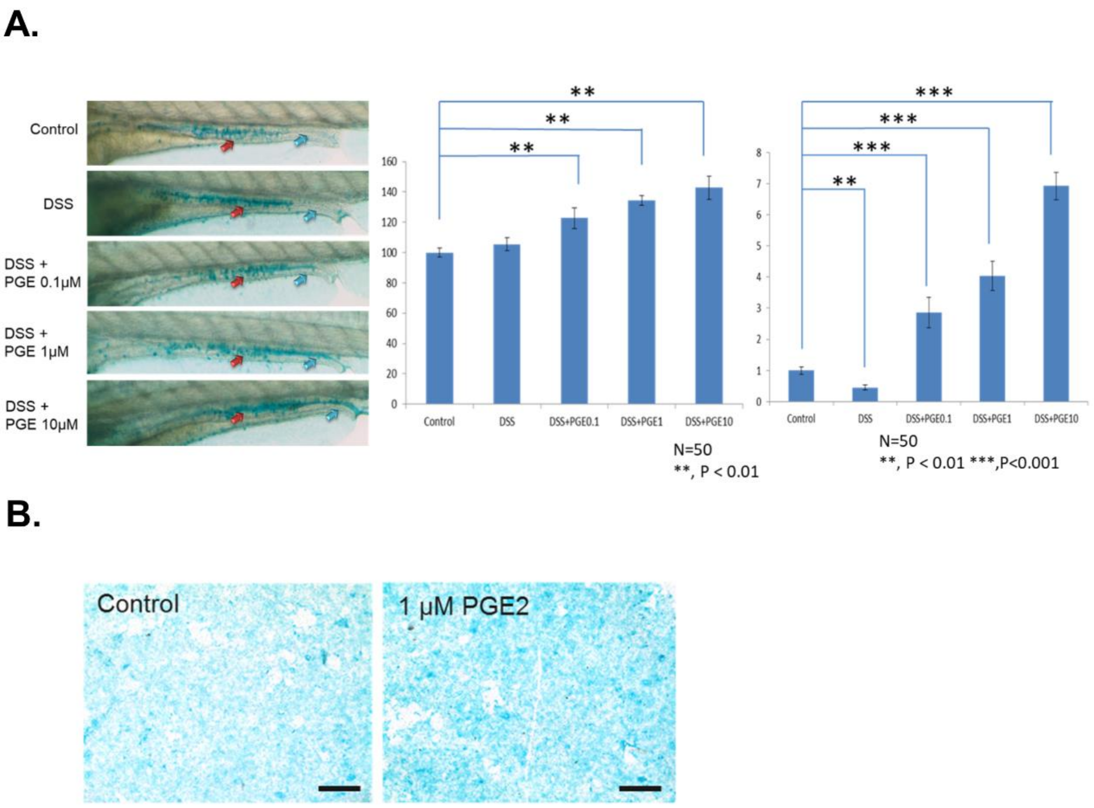

Fig. S12. (**A**) The images of Alcian blue staining with DSS alone or with treatments with 0.1, 1 and 10 μM PGE2 (left panel). The quantification of the whole gut region (red arrows) is shown in the middle panel, and the blue arrows indicate quantification of lumen areas (right panel). Scale bar, 100 μm. (N=100 from 3 clutches). **, P < 0.01. ***, P < 0.001. (**B**) Images of human enteroid-differentiated epithelium monolayer with and without PGE2 treatment. Scale bar 100 μm.

Disease Models & Mechanisms • Supplementary information

	Sequence (5' --> 3')	
**Gene**	**Forward**	**Reverse**
epcam	TTTGGATAAGAAACTTGTGTCTGAAG	TGTAGTACGGTCGTCCTTATCTTTTT
il1b	ATCAAACCCCAATCCACAGAGT	GGCACTGAAGACACCACGTT
mfap4	ATGGCAATCGTGCTGTTCTT	AACTTCTTGTGGCGTGTCA
mpeg1	TCACCTGCTGATGCTCTGCTG	TCTGTGGAATGACAAAGACCTC
muc2.1	CAACATCGATGGCTGCTTCTG	CTGACAGTAACATTCTTCCTCGC
p62	CGATGTTTTTGTCGGTCTCA	CAAGAGCCAAACCCATCATT
pllp	GGCCTTTGGTGCTGTTGGTAT	GCTGGCTGTGTATAACAGGGT
rppo	CTGAACATCTCGCCCTTCTC	TAGCCGATCTGCAGACACAC
tnfa	ACAACACTATTTACCTCGGC	ACCAAACACCCCAAAGAAGG

Table S1. Primers information for qPCR.

Disease Models & Mechanisms • Supplementary information

Supplementary movies

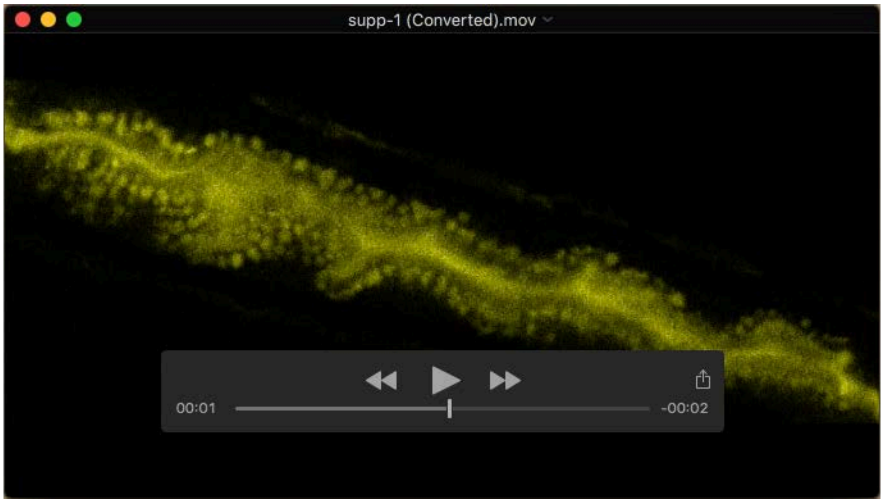

Movie 1. The confocal z-stack movies of the posterior mid-intestine of Tg(mfap4: turquoise) fish. Zebrafish treated with pH-rodo *E. coli* protein and co-staining with Cyto-ID for autophagy. The mfap4 positive cells show as yellow puncta.

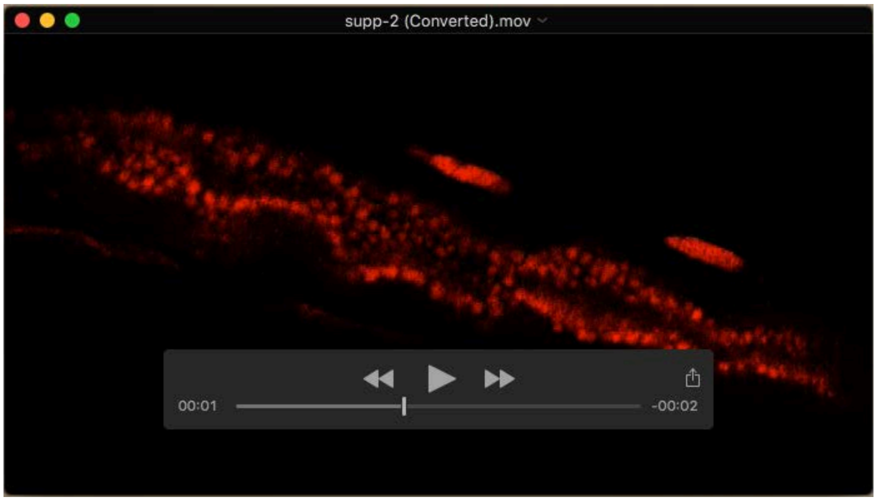

Movie 2. The confocal z-stack movies of the posterior mid-intestine of Tg(mfap4: turquoise) fish. Zebrafish treated with pH-rodo *E. coli* protein and co-staining with Cyto-ID for autophagy. The E. coli protein inside LRE cells shows in red puncta.

Disease Models & Mechanisms • Supplementary information

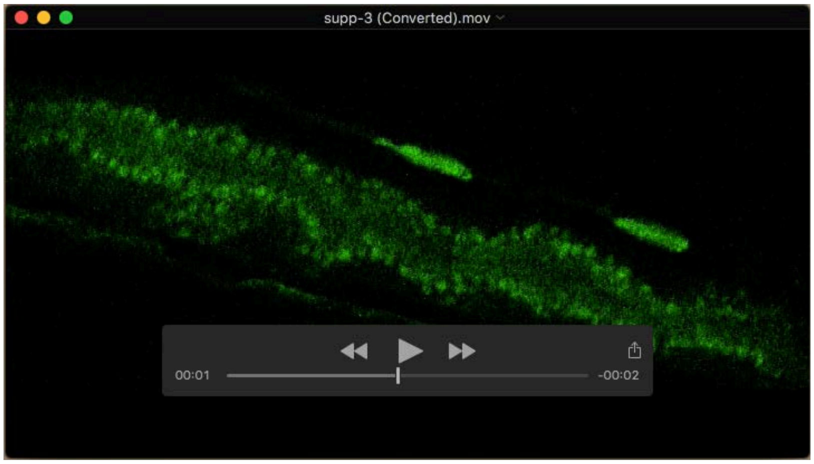

Movie 3. The confocal z-stack movies of the posterior mid-intestine of Tg(mfap4: turquoise) fish. Zebrafish treated with pH-rodo *E. coli* protein and co-staining with Cyto-ID for autophagy. The autophagy shows in green puncta.

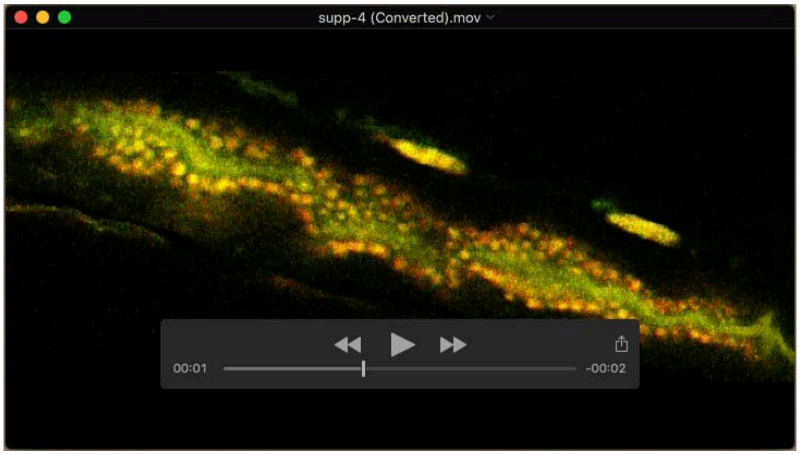

Movie 4. The confocal z-stack movies of the posterior mid-intestine of Tg(mfap4: turquoise) fish. Zebrafish treated with pH-rodo *E. coli* protein and co-staining with Cyto-ID for autophagy. A merged movie of S. movies 1-3 was shown. The three-color colocalization suggests ingested E. coli protein in the autophagy pathway.

Disease Models & Mechanisms • Supplementary information

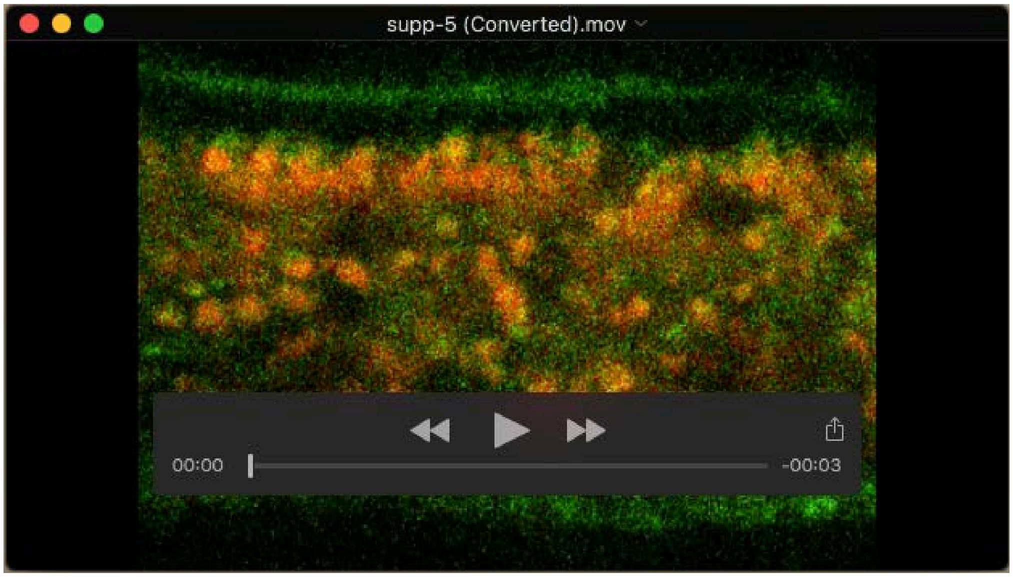

Movie 5. The confocal time-lapse movies of posterior mid-intestine in wildtype zebrafish. Zebrafish treated with pH-rodo *E. coli* protein in red color and co-staining with Cyto-ID for autophagy in green color. The red and green color both localize cells in the posterior mid-intestine over time.

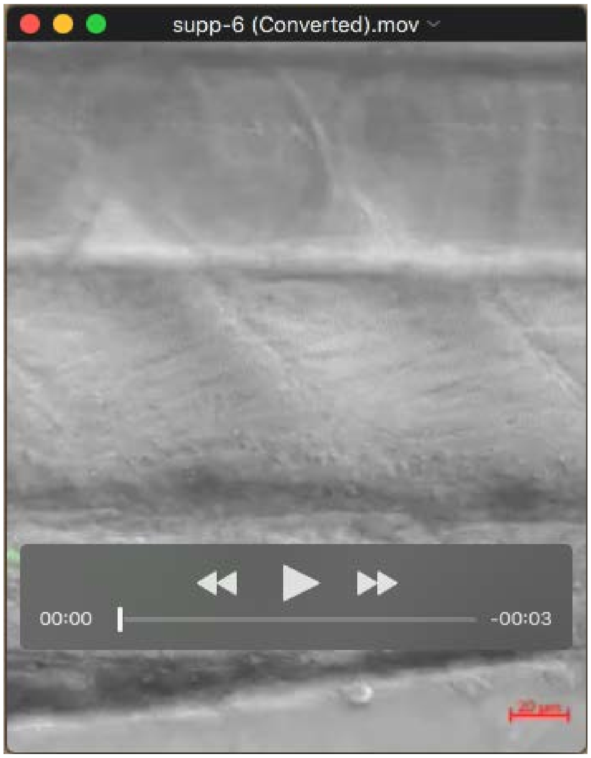

Movie 6. Bacterial invasion movie without DSS treatment. Green color indicates fluoresce Alexa-488 labeled heat-killed *E. coli* in the gut lumen but not dorsal aorta or cardinal vein.

Disease Models & Mechanisms • Supplementary information

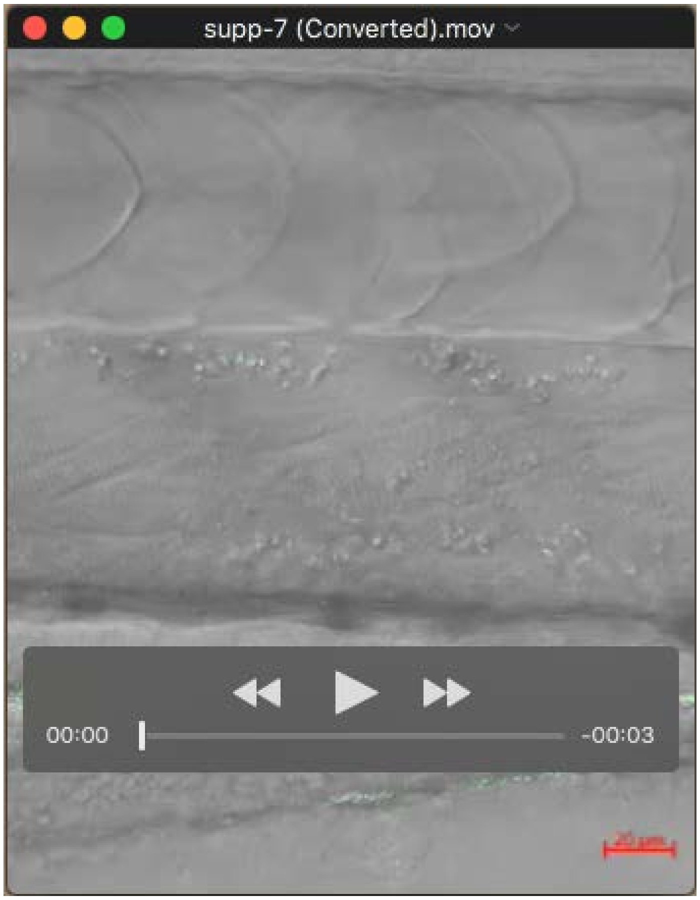

Movie 7. Bacterial invasion movie with DSS treatment. Green color indicates fluoresce Alexa-488 labeled heat-killed *E. coli* in the gut lumen, dorsal aorta, and cardinal vein.
